# Case Report: A challenging diagnosis of an apocrine sweat gland carcinoma

**DOI:** 10.3389/fsurg.2024.1307647

**Published:** 2024-03-20

**Authors:** Adeel Ahmad, Sajjaad Samat, Yaohong Tan, Harvey Bumpers

**Affiliations:** ^1^ Department of Surgery, Sparrow Health Systems, Lansing, MI, United States; ^2^ Department of Surgery, College of Human Medicine, Michigan State University, Lansing, MI, United States; ^3^ Department of Pathology, Sparrow Health Systems, Lansing, MI, United States

**Keywords:** apocrine carcinoma, sweat gland carcinoma, eccrine, apocrine, eccrine carcinoma

## Abstract

The differential diagnosis for an axillary mass in a patient with a previously treated malignancy is broad and definitive tissue diagnosis is required to guide treatment and surveillance strategies. We present the case of a 76-year-old African American male with a history of prostate cancer who presented with a left axillary mass two years after achieving remission from his prostate malignancy. Due to the diagnostic challenge, this excisional biopsy was reviewed at four different academic centers. Although no universal consensus among these institutions' pathologists, but in the context of clinical presentation and anatomic location, the overall clinical findings are consistent with apocrine sweat gland carcinoma. The mass was treated with complete local surgical excision, though regional lymph node metastasis occurred 2 years later. Multimodal treatment with surgery and radiation was done with removal of regional metastasis and no distant disease was identified. Primary apocrine carcinoma is a rare cutaneous neoplasm with less than 100 reported cases in the literature. A combination of clinical history and presentation, histomorphology, anatomical location, and immunohistochemistry is used to support the diagnosis and ultimately drive management.

## Introduction

Axillary masses present a diagnostic challenge due to the broad range of potential etiologies, including benign and malignant conditions. A thorough evaluation is crucial to differentiate between various possibilities and guide appropriate management. In this case report, we present the case of a 76-year-old African-American male with a history of prostate cancer who presented with a left axillary mass two years after achieving remission from his prostate malignancy, highlighting the steps taken to arrive at the rare diagnosis of apocrine sweat gland carcinoma.

Apocrine sweat gland carcinoma (ASGC) is an exceedingly rare malignancy arising from the sweat glands. While apocrine sweat gland tumors are uncommon, apocrine sweat gland carcinomas represent an even rarer subset, comprising less than 0.01% of all cutaneous malignancies (1, 2). These tumors pose diagnostic challenges due to their clinical variability and resemblance to benign conditions. In addition they have no specific immunohistochemistry profiles (3). Here, we present a unique case of apocrine gland carcinoma with atypical clinical features and an unexpected course of disease progression. By highlighting the diagnostic and therapeutic complexities encountered, this case report aims to contribute to the limited body of literature on this rare neoplasm and enhance our understanding of its clinical presentation, management, and prognosis.

ASGC typically arises from the apocrine sweat glands of the skin and can present as a slow-growing nodule or plaque. The tumor has the potential to infiltrate surrounding tissues, leading to local invasion and destruction. If left untreated or inadequately managed, ASGC can progress to involve regional lymph nodes and metastasize to distant sites, including the lungs, liver, bones, and brain (3).

Recurrence rates following surgical excision of ASGC vary widely, ranging from 20% to 65%, depending on factors such as tumor size, depth of invasion, and the presence of lymphovascular invasion. The risk of metastasis is also relatively high, with reported rates ranging from 15% to 50%. Lymph node involvement is a strong predictor of distant metastasis and overall survival (3).

ASGC can behave aggressively, and its prognosis is generally poor compared to other cutaneous malignancies. The survival rates vary widely depending on tumor stage, histological type, and distant metastasis (3). The ten-year survival rate has been reported to be around 56%, decreasing to 9% with positive lymph node findings (3, 4). Since these tumors are rare and with a small body of literature there has been no significant change in management or survival (5, 6). Prompt diagnosis, accurate staging, and appropriate management strategies including wide surgical excision, lymph node assessment, and adjuvant therapy (such as radiation or chemotherapy) when indicated, are important for optimizing patient outcomes.

## Case presentation

The patient is a 76-year-old African-American male with a past medical history of hypertension, hyperlipidemia, and prostate cancer that was treated with radiation therapy in 2017. He presents with a self-identified left axillary mass in June 2020. He denies constitutional symptoms of fever, fatigue and weight loss. He is otherwise asymptomatic. Physical exam demonstrates a 1.5 cm × 2 cm mobile, non-tender inferior axillary mass. Core needle biopsy in August 2020 demonstrated an adenocarcinoma which was weakly positive for NKX3.1 (NK3 homeobox 1), a prostate adenocarcinoma marker with nuclear staining, though indeterminate due to weak expression (7). It was negative for PSAP (prostatic acid phosphatase), TTF1 (thyroid transcription factor 1), CK7 (cytokeratin 7), CDX2 (caudal-related homeobox gene 2), p63 (tumor protein 63) and CK20 (cytokeratin 20). Due to weak positivity of NKX3.1 and history of prostatic adenocarcinoma, metastatic prostatic carcinoma was favored. In order to have a more definitive diagnosis, this case was sent out for a second opinion at a neighboring academic institution which supported the diagnosis of a poorly differentiated adenocarcinoma with non-specific immunophenotyping. Again, PSA expression was found to be negative and NKX3.1 was reported to be patchy.

**Figure 1:**
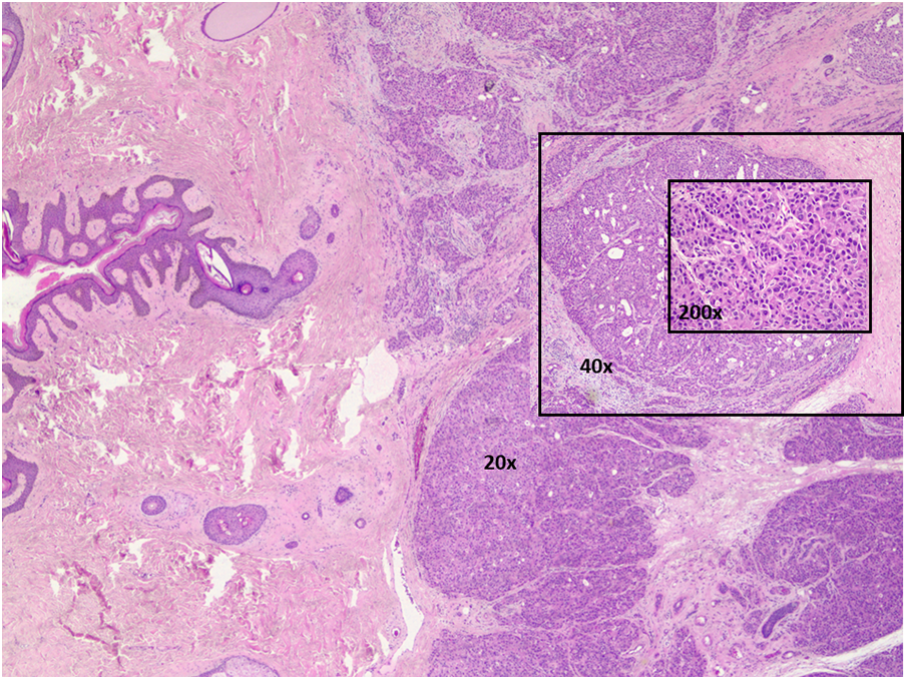
Apocrine sweat gland carcinoma is moderate to poorly differentiated. Hematoxylin & eosin stained. 20×, 40×, and 200× magnifications.

Due to the uncertainty regarding oncological recurrence, the decision was made to proceed with excisional biopsy. Our patient underwent excision in September 2020. Histologic examination of the entire specimen revealed a large multilobated tumor involving the dermis and superficial subcutis. The tumor cells are large and epithelioid with abundant eosinophilic cytoplasm, forming solid sheets with focal glandular architecture, but no secretion (Figure 1). With a large panel of immunostains, the tumor is weakly positive for GATA3 (GATA-binding protein 3), NKX3.1, ER (estrogen receptor), BRST (same as GCDFP-15) and CK20, patchy staining, and is negative for all other markers that were studied: PSA (prostate specific antigen), PSMA (prostate specific membrane antigen), CK7 and p63. This case was reviewed at four major academic institutions. Though based on histopathology there was no universal consensus among pathologists at these institutions, most of them agreed with the diagnosis of apocrine sweat gland carcinoma. This was based on histomorphology, immunophenotypic profile, anatomic location and clinical presentation. Unfortunately a margin was positive in the initial excision, so the patient underwent an additional marginectomy in November 2020.

Because margins were negative following the marginectomy in November, no additional intervention was made and the patient was instructed to conduct routine surveillance for local recurrence.

**Figure 2:**
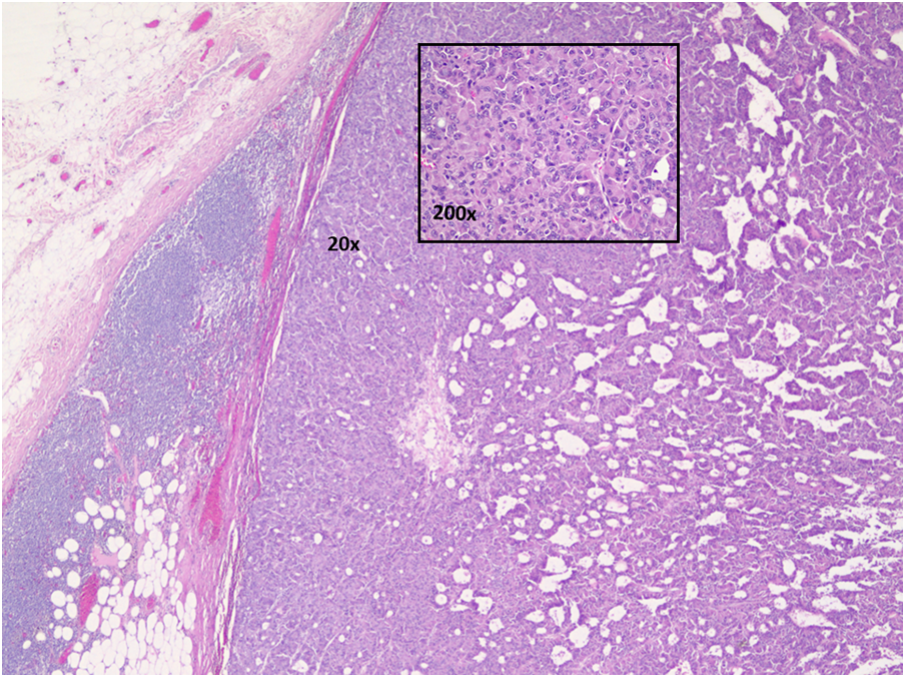
Axillary lymph node with metastatic carcinoma. Hematoxylin and eosin stained. 20× and 200× magnifications.

Two years later, the patient returned in October 2022 with a new left axillary lump. He underwent an ultrasound-guided core biopsy that demonstrated an adenocarcinoma with similar morphology (Figure 2) and immunoprofile as those of the apocrine sweat gland carcinoma in the original axillary mass in 2020.

Our patient underwent a PET scan in December 2022 which demonstrated a single hypermetabolic nodule in the axilla measuring 2.5 cm × 1.5 cm. He was taken to the operating room for a left axillary lymph node dissection later that month. Pathology demonstrated metastatic carcinoma in one of twelve lymph nodes. The carcinoma has similar morphology and immunoprofile to those of apocrine sweat gland carcinoma. He was referred to the radiation oncologist for adjuvant radiation therapy. The plan was continued surveillance for local recurrence following definitive therapy. To this date the patient has not had any signs or symptoms of recurrence.

## Discussion

The identification of a new axillary mass in a patient with a previously treated malignancy creates a broad differential diagnosis. Definitive diagnosis is critical to guide treatment and surveillance strategies.

Immunohistochemistry staining plays an important role in identifying tumor markers which may identify the primary tissue of origin. However, it may cause confusion sometimes.GCDFP-15 (gross cystic disease fluid protein-15) and PSA are markers associated with breast and prostate glandular epithelium, respectively (8, 9). In this case, the tumor persistently stains positive for NKX3.1, though weak. NKX3.1 is a prostatic tumor suppressor gene which often stains weakly in high grade prostate cancer and does not stain at all in most metastatic prostate cancers (10). Although it has a very high specificity, but weak expression has also been seen in tissue of other organs. This makes weak staining very nonspecific. The other markers that are specific for prostate, including PSA and PSAP, are all negative in this tumor. GATA3 is not a specific lineage defining marker, and is positive in many tissue types including breast, urothelium, skin and adnexal tumors. Therefore, correlation with clinical findings and histomorphology is very important in evaluating the immunostaining results. This case presented such a challenge to diagnosis because apocrine sweat gland carcinoma is so rare, and it has no specific immunoprofile. Also the patient's not very distant history of prostate cancer caused confusion. Given the lack of a specific immunoprofile for apocrine sweat gland carcinoma it was necessary to use multiple factors to derive this diagnosis: histomorphology, immunophenotype, anatomical location, and clinical profile.

The management of apocrine sweat gland carcinoma involves a multidisciplinary approach. Surgical excision with wide margins is the mainstay of treatment for localized disease. The extent of surgery depends on factors such as tumor size, location, and depth of invasion. In cases where lymph node involvement is suspected or confirmed, lymph node dissection or sentinel lymph node biopsy may be performed to assess the spread of the disease. This is unchanged since the early treatment of axillary metastatic disease with radical axillary dissection in 1955 (5). At that time there had only been 18 cases of sweat gland carcinoma with metastasis reported in the literature since its initial description in 1911 by Hedinger (5, 11). Adjuvant therapies, including radiation therapy and chemotherapy, are considered based on the individual patient's risk factors, tumor characteristics, and presence of metastatic disease (12). However, In our patient with only 1 of 12 lymph nodes positive for metastasis, a PET scan negative for distant disease, and the poor effect of chemotherapy on this cancer (12) chemotherapy was not recommended. Due to the rarity of ASGC and limited evidence from clinical trials, optimal management strategies have not been firmly established. In conclusion, a personalized treatment plan should be developed in collaboration with a multidisciplinary team, taking into account the specific characteristics of the patient and the rare apocrine sweat gland tumor.

## Data Availability

The original contributions presented in the study are included in the article/Supplementary Material, further inquiries can be directed to the corresponding author.
